# An approximate Bayesian approach for mapping paired-end DNA reads to a reference genome

**DOI:** 10.1093/bioinformatics/btt073

**Published:** 2013-02-14

**Authors:** Anish Man Singh Shrestha, Martin C. Frith

**Affiliations:** Computational Biology Research Center, National Institute for Advanced Industrial Science and Technology (AIST), Koto-ku, Tokyo 135-0064, Japan

## Abstract

**Summary:** Many high-throughput sequencing experiments produce paired DNA reads. Paired-end DNA reads provide extra positional information that is useful in reliable mapping of short reads to a reference genome, as well as in downstream analyses of structural variations. Given the importance of paired-end alignments, it is surprising that there have been no previous publications focusing on this topic. In this article, we present a new probabilistic framework to predict the alignment of paired-end reads to a reference genome. Using both simulated and real data, we compare the performance of our method with six other read-mapping tools that provide a paired-end option. We show that our method provides a good combination of accuracy, error rate and computation time, especially in more challenging and practical cases, such as when the reference genome is incomplete or unavailable for the sample, or when there are large variations between the reference genome and the source of the reads. An open-source implementation of our method is available as part of Last, a multi-purpose alignment program freely available at http://last.cbrc.jp.

**Contact:** martin@cbrc.jp

**Supplementary information:** Supplementary data are available at *Bioinformatics* online.

## 1 INTRODUCTION

**Fig. 1.**
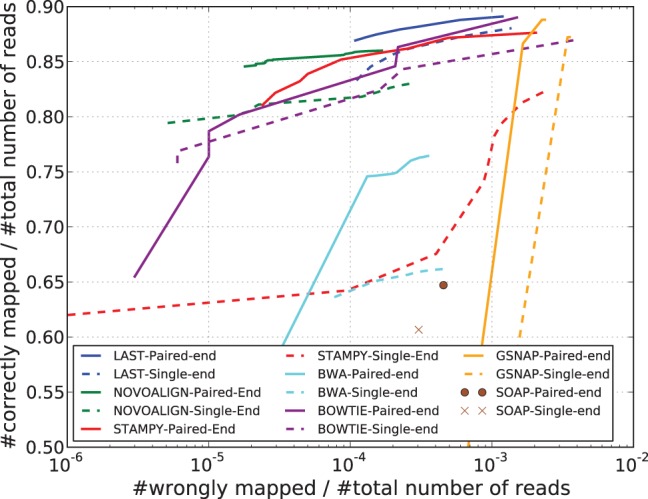
Paired-end versus single-end mode. Comparing the results of mapping the same set of simulated 100 bp-long human reads with the human reference genome hg19, in paired-end mode (solid lines) and single-end mode (dashed lines). The caption of Figure 3 describes how the lines in the plot above are computed. Details of the simulation process are provided in Section 3.1

Many high-throughput sequencers provide a paired-end option, in which each of the two opposite strands of a DNA fragment is read from the edge to the interior in the 5′–3′ direction, generating a pair of reads. Paired-end reads can be obtained by a simple modification to the standard single-end workflow; yet, they provide several benefits over single-end reads. They contain extra positional information that aids in accurate mapping of reads to a reference, for instance, by disambiguating alignments when one of the ends aligns to a repetitive region. They are also extremely useful in downstream analyses of structural variations, such as detection of indels or rearrangements. In this article, we focus on the former: the task of mapping a set of paired-end reads to a reference genome, which is often the first and fundamental step in inferring biological phenomena from high-throughput sequencing data. To motivate our work, we compare in Figure 1 the results of mapping 1 million pairs of simulated human reads to the human reference using various alignment tools in their paired-end and single-end modes. For nearly all the aligners, the use of pairing information significantly improves mapping accuracy.

Various read mapping tools support paired-end data, although not all provide adequate statistical treatment. Mappers such as Bowtie2 (Langmead and Salzberg, 2012), SOAP2 (Li *et al.*, 2009) and GSNAP (Wu and Nacu, 2010) require the user to input the expected value and standard deviation of the genomic distance between the two ends. Based on this information, they flag a pair of mapped reads as being either ‘concordant’ or ‘discordant’, for possible use in analyses of structural variations. However, the user may not know before-hand the expected genomic distance, especially when relying on third-party datasets—some provide reliable information about the fragment size distribution, but not all do. BWA (Li and Durbin, 2009) does not rely on user-provided fragment size thresholds and estimates the fragment size distribution from uniquely mapped pairs. Another feature of the paired-end strategy of BWA is that if only one end is reliably mapped, it attempts to ‘rescue’ the other end by aligning it using the Smith–Waterman algorithm in the area implied by the inferred fragment size. Like BWA, STAMPY (Lunter and Goodson, 2011) learns the fragment size distribution from the input data and also attempts to rescue mates for pairs whose ends do not map uniquely and at sufficiently close distance to each other. Additionally, it uses an elaborate probabilistic model that incorporates pairing information to compute for a pair of alignments of a read pair, the posterior probability of having predicted an incorrect alignment. Novoalign (www.novocraft.com) computes a similar mapping quality for each paired alignment, but it relies on the user to provide the fragment size distribution.

We propose a new approach to apply pairing information for mapping paired-end reads. We first align each read independently to obtain candidate alignments. For each candidate, we apply our new probabilistic model to estimate the posterior probability that this alignment is incorrect by considering other candidate alignments of the read, those of its mate and the fragment size distribution. To allow for the possibility that paired reads may actually come from disjoint locations in the reference, our model includes a prior probability on the occurrence of such an event. One major departure from previous methods is that we calculate marginal posterior probabilities for each candidate alignment of a read (as opposed to associating this value to a read pair). Our method also benefits from using Last (Kiełbasa *et al.*, 2011) in the alignment phase. Last, like BLAST, computes local alignments based on a seed-and-extend technique, but it is fast because of the use of adaptive seeds—thus allowing the application of classic sequence alignment techniques to the problem of mapping giga-scale sized sets of reads generated by high-throughput sequencers. We describe our method in detail in Section 2.

We compare the performance of our method with six other mapping tools in Section 3. For each mapping tool, the technique used to align individual reads hugely influences the final outcome, and as it is not feasible to isolate the pairing algorithm, we are comparing to some extent the performance of the alignment algorithms as well. Many articles that introduce new read mapping tools or survey them provide benchmarking tests that tend to be not very informative—only ideal or easy cases are tested—or even misleading—performance is measured by counting the number of reads mapped, with complete disregard to the correctness of mapping. In this article, we conduct more rigorous tests that cover various practical scenarios.

## 2 PROPOSED METHOD

Our method comprises the following steps.

### 2.1 Outline

1. Perform local alignment between the genome and each read individually, and keep alignments that have score higher than a threshold.
2. Using these alignments, estimate the distribution of fragment lengths.
  - For each read pair, get all pairs of alignments to opposite strands of the same chromosome. For every such alignment pair, infer the fragment length. If the read pair has exactly one distinct fragment length, record it. Note that we do not impose any bound on the size of the inferred fragment.
  - Find the sample median and quartiles of the fragment lengths.
  - Assume the fragment lengths are normally distributed, with mean = sample median and standard deviation = interquartile range/1.34898. We use this method of estimation as it is robust to outliers. (1.34898 is the interquartile range of a normal distribution with standard deviation of 1).
3. Estimate the probability that each alignment represents the genomic source of the read, using a probabilistic model.

### 2.2 Probabilistic model

We shall now describe Step 3 in detail. Consider a pair α and β of reads obtained from sequencing two ends of a DNA fragment, and let *a*, *b* be a pair of predicted alignments of α and β, respectively, to the reference. Let *d* be the prior probability that a read pair comes from disjoint genomic locations. This might arise from real differences between the reference and the source of the reads, or from errors in obtaining the reads or the genome sequence. The value of *d* can either be provided by the user or learned from the data once the fragment length distribution has been estimated (in our experiments, we use a default value of 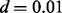). If we assume that a pair comes from any location in the reference with uniform probability, we can express the prior probability as:

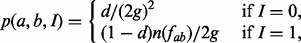

where *I* serves as an indicator variable (0 for reads being disjoint, 1 for conjoint), 2 *g* is the number of bases in both strands of the haploid genome, *f_ab_* is the fragment length implied by *a* and *b* and 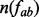 is the probability of fragment length based on the distribution estimated in Step 2.

Next, we wish to compute the likelihood 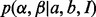 of having observed α and β given alignments *a* and *b*. Given an alignment between a pair of sequences, classical sequence alignment methods assign it a score, which is a measure of the likelihood that the sequences are related as opposed to being unrelated (Durbin *et al.*, 1998). This is done based on a scoring model, which assigns a score *S_xy_* for aligning a pair of bases 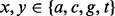. *S_xy_* can be interpreted as a log likelihood ratio:

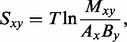

where *A_x_* is the probability (abundance) of base *x* in the reference, *B_y_* is the probability of *y* in the query, *M_xy_* is the probability of *x* aligned to *y* in a true alignment and *T* is an arbitrary scale factor. This scoring model can be generalized to include gaps (Durbin *et al.*, 1998) and also to incorporate sequence quality data (Frith *et al.*, 2010). As Last is based on this generalized scoring model, the score assigned by Last to an alignment is the log of its likelihood ratio. Therefore, if *s_a_* and *s_b_* are the scores of alignments *a* and *b* and *T* is the scale factor, by exponentiating the scores, we arrive at:

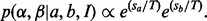



Finally, letting *A* and *B* denote the set of all possible alignments of α and β to the reference, we can calculate the posterior probability 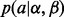 of *a* indicating the true genomic source of the read as follows:
(1)
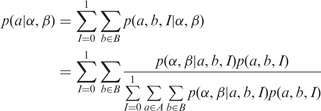



Naturally, it is not feasible to exhaustively search *A* and *B*; therefore, we approximate (1) by restricting *A* and *B* to be the set of alignments that are produced by Last in Step 1.

### 2.3 Efficient computation

To calculate the posterior probabilities efficiently, we use the following intermediate values:

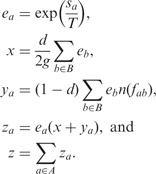



Then Equation (1) can be rewritten as:
(2)
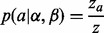



### 2.4 Accounting for alignment cut-off

Step 1 inevitably uses a cut-off: it returns alignments with score 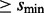. This may lead to false-positive mappings.

For example, suppose we have a read pair that is truly conjoint, where the first read’s true alignment has score 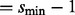, and the second read’s true alignment has score 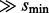. Suppose the first read has a random alignment with score 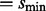. Our probabilistic method will confidently predict an incorrect mapping.

To solve this problem, we modify our probabilistic method slightly. We assume an unlucky case: the first read has an alignment with score 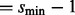, at the optimal distance from the best alignment of the second read (as we use integer scores, 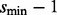 is the highest possible score 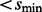). Let 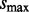 be the maximum score of any alignment in *B* and 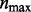 be the maximum value of *n*(*f*) for any *f*. We modify Equation (2) as:

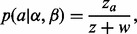



where

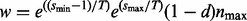



### 2.5 The case where 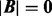


The preceding calculations do not work when 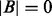. In this case, we define the result to be the same as if there was one alignment in *B* on a different chromosome to any alignment in *A*.

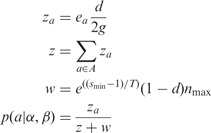



## 3 PERFORMANCE TESTING

In this section, we compare the results of testing our method with those of other mapping tools that provide a paired-end option. We tested the following: Novoalign, Stampy, BWA, Bowtie, GSNAP and SOAP. Although the list is not comprehensive, the tools chosen are representative of the different paired-end mapping strategies discussed in Section 1 that have been used so far to deal with paired-end reads. Obviously the performance is also hugely affected by how individual reads are mapped because this phase precedes the pairing phase for most aligners. With our selection of mapping tools, we have also attempted to cover a wide range of techniques that are used for mapping individual reads. BWA, Bowtie and SOAP use the Burrows–Wheeler transform to index the reference, whereas Novoalign, Stampy and GSNAP use hash-bashed techniques. BWA, SOAP and GSNAP search for semi-global alignments that contain no more than a certain user-specified number of mismatches and gaps, whereas Stampy and Novoalign follow the traditional seed-and-extend technique with affine gap penalties. Newer versions of Bowtie and BWA can perform both semi-global, as well as local alignments. The version information of each aligner is provided in the Supplementary Material.

We conducted a series of tests that cover a variety of practical scenarios, such as when the reference is incomplete, when the sample lacks a reference and has to be mapped to the reference of a close species or when there are large variations between the reference and the sample that have the effect of confounding the pairing relation.

Our results, which we describe later in the text, show that Last provides a good combination of sensitivity, error rate and computation time and is consistently among the top performers in all of the tests. However, we realize that each aligner comes with numerous parameter settings that provide a trade-off among sensitivity, error rate and time, and it is difficult to judge performance based on a single set of parameters. Here, we have tried to show the aligners in the best light possible. For most aligners, we report the best results after having tried various parameter settings, details of which can be found in the Supplementary Material. With Last, we use the same settings throughout all the tests so as to avoid deliberate optimization to fit the data.

### 3.1 Generation of simulated data

**Fig. 2.**
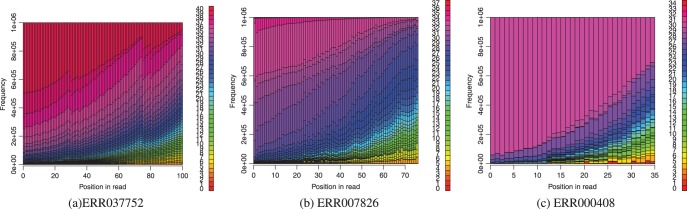
Error profiles of the first 1 million reads of the real datasets based on which we have simulated sequencer errors. The horizontal axis shows position along the 5′–3′ direction along the read, and the vertical axis shows for each position the frequency of reads based on their phred-scaled quality scores

Although it is desirable to work with real datasets, they are accompanied by the problem of not knowing the true genomic location of the reads. This limits us to working with simulated data. Using Dnemulator (www.cbrc.jp/dnemulator), a package for simulating DNA sequencing errors and polymorphisms, we generated paired-end reads from chromosomes 1–22 and X of the human genome hg19. Starting with hg19, we simulated a diploid genome by incorporating polymorphisms into it. This was done by picking real alleles based on their frequencies obtained from snp132Common.txt, a SNP database available from the UCSC Genome Database (Fujita *et al.*, 2011, Sherry *et al.*, 2001). Next, from the simulated genome, we randomly drew a million fragments from which paired-end reads of length 100 bp were generated. Finally, we simulated sequencer errors, according to the per-base error probabilities of the first million pairs of reads in ERR037752, downloaded from DDBJ. In a similar manner, simulated paired-end reads of lengths 76 and 35 bp were also generated with error profiles based on datasets ERR007826 and ERR000408, respectively. The error profiles of these three datasets are shown in Figure 2. The Supplementary Material includes further details about the simulation workflow.

Additionally, we generated a second 100 bp-read dataset using the same 100 bp reads as aforementioned, but simulating sequencing error based on error probabilities from SRR067577. As SRR067577 contains significantly more low-quality reads than ERR037752, this dataset allowed us to understand the effect of error profile on aligner performance. All experiments we describe below were also repeated on this dataset. Because of space constraints, we provide those results in the Supplementary Material.

### 3.2 Mapping simulated human reads to human reference

**Fig. 3.**
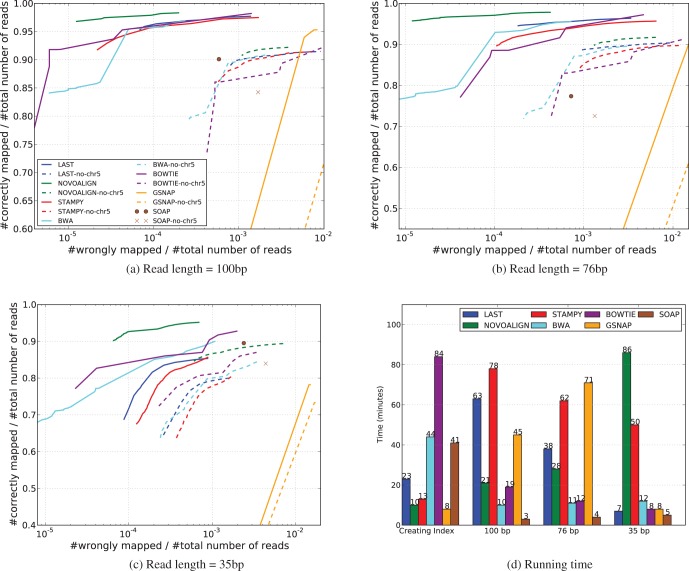
(**a–c**) The result of mapping 1 million pairs of simulated short reads of various lengths to Chromosomes 1–22 and X of hg19, respectively. Most aligners assign to each reported alignment a mapping quality score, which reflects the aligner’s estimate of the probability that the alignment is incorrect. Although Last reports raw probability values, some aligners like BWA and Bowtie apply phred-like scaling to obtain discretized integer scores. In either case, the mapping results from an aligner can be filtered to obtain only those alignments that pass a certain mapping quality threshold. Each curve in the plots above is obtained from connecting discrete points, each point corresponding to the fraction of wrongly mapped and the fraction of correctly mapped reads at a certain mapping quality threshold. When varying the mapping quality threshold, we were careful not to go below the mismap probability of 0.5. As it is not possible to have more than one alignment with mismap probability <0.5, this avoids the complications of having to evaluate cases of multiple/secondary mappings. Solid lines are for mapping reads to a complete reference, and dashed lines are for the test with reference with chromosome 5 missing. An exception is SOAP, which does not provide mapping quality values; therefore, it is represented by a single data point in each plot. (**d**) The running time for creating the index of the reference genome and the alignment time corresponding to the results of (a–c), when executed on a single core of a machine with Intel(R) Core(TM) i7-3770K CPU @ 3.50 GHz and 32 GB random access memory

To start with a relatively easy test, we mapped our three sets of simulated reads to the human reference genome containing Chromosomes 1–22 and X of hg19. For aligners that require the user to input the fragment length statistics, we provided the exact values used to produce the simulated dataset. The results are shown in Figure 3 by solid (non-dashed) lines.

For the dataset containing reads of length 35 bp, edit-distance–based aligners outperform Last. For longer reads, Last is among the top performers. Novoalign is consistently the best aligner, but its run time gets extremely large for short reads.

To see how sequencer error rate affects the results, we repeated this experiment with the extra 100 bp dataset. The results (Supplementary Fig. S4a) show that Last does better than all the other aligners when the reads are more error-ridden. The results in Figure 1 were also obtained using this dataset. And while Last does not require explicit trimming of reads, failing to do so can significantly worsen the performance of some aligners like BWA and SOAP. We address this issue of read trimming in greater detail in the Supplementary Material.

Finally, one concern about Last might be that the run time for longer reads is relatively slow. We describe in Section 4.2 several techniques to make Last achieve higher speeds without degrading accuracy.

### 3.3 Mapping to an incomplete reference

Because of incompleteness of sequence assembly, the available reference genome may have large chunks missing. Some reads possibly come from regions that are missing in the reference, confounding both the alignment algorithm, as well as the pairing algorithm. To simulate this scenario, we repeated the earlier experiment with the same set of simulated reads, but with a part of the reference (Chromosome 5 was chosen arbitrarily) deliberately removed. The results are shown in Figure 3 by dashed lines.

As Chromosome 5 accounts for ∼5% of the full reference, it is natural that the number of correct mappings decreases by about the same proportion. It is interesting, however, that the error rate also increases, even for Novoalign, which showed very low error rates in the test with the full reference.

### 3.4 Xenomapping

**Fig. 4.**
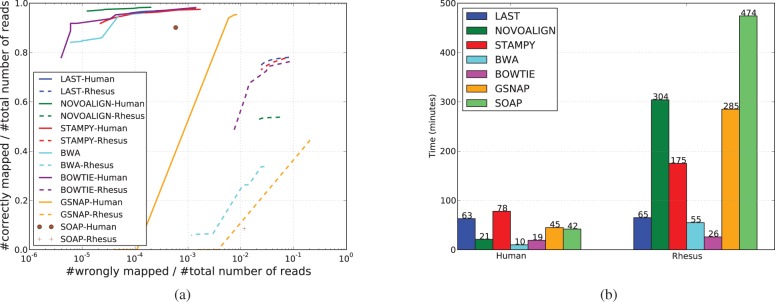
Xenomapping. (**a**) The result of mapping 1 million pairs of simulated human reads of length 100 bp to rheMac2 (dashed lines) compared with that of mapping the same reads to hg19 (solid lines). (**b**) The running time (excluding the genome indexing step) corresponding to the results of (a)

A great majority of organisms lack a reference genome. Even for mammals, of which there are >5000 species, only a small number of them have been sequenced. For samples with no available reference, reference of a related species is used. This is also the case for extinct species. While divergence between the two genomes can make the task of aligning individual reads harder, it can also make the pairing phase more challenging, as a pair of reads may map to disjoint locations because of rearrangements in the two genomes since their last common ancestor. To simulate this situation, we map our simulated human reads to the rhesus monkey reference (rheMac2). To decide the correctness of reported alignments, we use the human–rhesus pairwise alignments (rheMac2.hg19.all.chain) provided by UCSC. In doing so, we are assuming that the alignments provided by UCSC are correct, which might not always be the case; however, we can afford to neglect this issue, as it affects all mappers equally. The results are shown in Figure 4.

Last is again among the top performers, alongside Bowtie, which was used in the local alignment mode, and Stampy. As would be expected, edit-distance–based methods like BWA, GSNAP and SOAP perform poorly because the expected number of mismatches between the read and reference is high. The run times, shown in Figure 4b, are also worth noting. Most of the other aligners get drastically slow compared with their run times for mapping human reads to human reference. A similar trend was observed for another dataset of read length 100 bp (Supplementary Fig. S4b).

### 3.5 Chromosome translocations

**Fig. 5.**
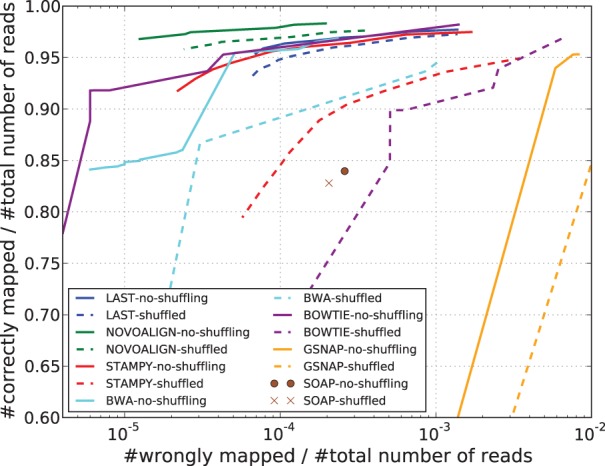
Result of mapping 1 million pairs of simulated human reads of length 100 bp with 40% of reads in one of the fastq files shuffled (dashed) compared with no shuffling (solid)

Even within the same species, real differences between the source and reference genomes can arise because of chromosomal abnormalities in the source or the reference that are caused by rearrangements of segments of chromosomes. This phenomenon of translocations is a common occurrence, for instance, in cancer cells. If a pair of reads happens to span a translocation break point, the two ends might be disjoint in the reference. This could possibly confuse the pairing algorithm. To test whether mappers are tolerant to this kind of phenomenon, we randomly shuffled a portion (40%) of the reads in one of the fastq files, thus re-assigning those pairs. Although this does not truly reflect the natural process of chromosomal translocations, it does have a similar effect of dismembering paired reads. Also, although 40% of the pairs being disjoint is unrealistic even for, say, cancer genomes, the main interest of many studies lies precisely in these reads. Therefore, it is imperative that aligners be able to map these reads accurately, and the objective of this test was to see how well this is done. Figure 5 shows the result of mapping these shuffled reads compared with the result of Section 3.2. We can see that while Last and Novoalign are only slightly affected by shuffling, the remaining aligners see a sharp decrease in accuracy. Repeating this experiment on our second 100 bp dataset yielded similar results (Supplementary Fig. S5). It must be pointed out that in this experiment, we have left the setting of *d*, the prior disjoint probability, to its default value of 0.01. We describe in Section 4.5 and the Supplementary Data how choosing the value of *d* wisely can improve Last’s performance.

### 3.6 Testing with real data: flow-sorted human X-chromosome

**Fig. 6.**
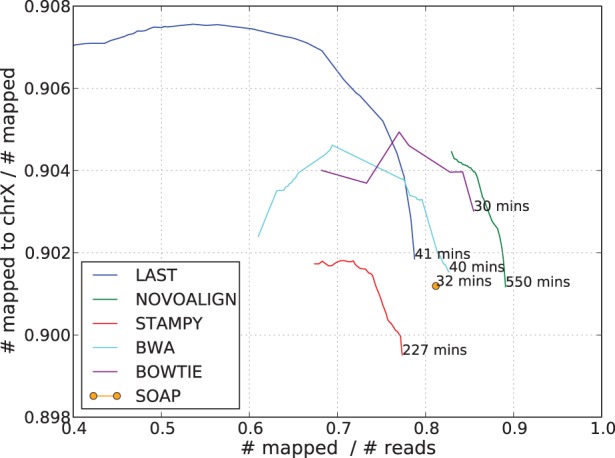
Result of mapping 2 703 583 pairs of 35 bp-long reads from DDBJ dataset ERX000112 to Chromosome 1–22 and X of hg19. The horizontal axis shows the fraction of reads that were mapped, and the vertical axis shows the fraction of the mapped reads that were mapped to the X chromosome. The figure at the bottom end of each curve indicates the running time (excluding time for genome indexing) in minutes

Simulation may miss some aspects of real data; therefore, it is desirable to work with real datasets. Real data means lack of knowledge of true genomic locations of reads. We worked around this problem by using paired-end reads (DDBJ dataset ERX000112) of flow-sorted X chromosomes. This dataset contains 2 703 583 pairs of reads of length 35 bp. As we know that the reads come from the X-chromosome, we can treat the reads mapped to a chromosome other than X as being wrongly mapped. Results of mapping these reads to chromosomes 1–22 and X of hg19 are shown in Figure 6. That the results closely resemble those in Figure 3c works as a validation of our simulation technique. Certainly, we must take into account possible contamination of DNA from non-X chromosome in the dataset. The experiment metadata claims 90% purity, which we corroborated by using Last in a slow-but-sensitive mode that resulted in 9–10% of the mapped reads being mapped to non-chromosome X.

## 4 DISCUSSION

### 4.1 Simulation limitations

Our simulation fails to capture some real-world details. Our method of simulating sequencer errors based on the per-base error probability reported in experimental datasets is more realistic than previous methods of introducing uniform random errors. However, we assume that the reported probabilities are correct, which might not always be true depending on the sequencer and the experiment. Also, we do not consider adapter sequences, which in real experiments might be attached to reads. Furthermore, we have assumed that our read sets are free of contaminants or artifacts, which is unlikely with real data.

### 4.2 Speeding up Last

Applying several simple workarounds, we can significantly improve the speed of Last without degrading its accuracy. The alignment phase of Last takes approximately two-thirds of the total running time. Last performs gapless alignments significantly faster than gapped alignments; for cases where the sample and reference have few indel polymorphisms between them, Last can be used in the gapless mode. We show in the Supplementary Material that mapping human reads to the human reference in a gapless mode reduces the running time of the alignment phase by >50% with no cost to accuracy.

The pairing phase can also be made faster. As the paired-end module is written in python, file operations can be costly. As its default output file format, the alignment phase uses maf format. Changing this to the smaller tabular format can significantly bring down the running time of the pairing phase by 30% (Supplementary Data). Also, greater speed can be achieved by running the paired-end script using a faster implementation of Python, such as PyPy. The running times reported in this article were obtained when using PyPy. Another way to speed-up the pairing phase is to quickly estimate the fragment size distribution using a large-enough sample instead of using the complete set of aligned reads, and then apply this estimate to calculate the mismap probabilities.

Avoiding temporary files by using named pipes also avoids speed issues due to slow disk access operations.

These speed-up techniques are discussed in the Supplementary Material and also appear in the user manual provided with the program.

### 4.3 Extension to RNA

Last allows mapping of paired-end RNA reads. To handle RNA data, we modify the fragment length distribution model and the prior disjoint probability. We observed from a test with human RNA-seq data that the distance distribution resembles a mixture of two log-normal distributions, having a prominent peak for shorter introns and a much smaller peak for long introns. For simplicity, we assume that the genomic fragment lengths of the reads come from a single log-normal distribution. To compensate for not incorporating longer introns in the model, the default disjoint probability value is increased from its default value of 0.01 to 0.02.

### 4.4 Circular DNA

Many prokaryotic cells as well as eukaryotic organelles contain circular chromosomes. With circular chromosomes, we must ensure that we do not consider as disjoint a pair of reads that straddle the position at which the circular sequence is disrupted to obtain its linear representation. Last provides an option to specify whether any chromosomes in the reference are circular.

### 4.5 Learning prior disjoint probability

In our tests, we have used the default value for the prior probability of a read pair being disjoint. Instead of relying on a default value, it is possible to estimate it from the set of uniquely mapping reads. Our experiments with setting this value to match the actual proportion of disjoint pairs show that it slightly improves accuracy. However, we are yet to implement this feature in the program. In contrast, changing a similar prior probability setting of Stampy brought about no significant changes in performance.

## 5 CONCLUDING REMARKS

In this article, we have shown that Last is a versatile aligner, and it is especially promising for ‘hard’ cases. We believe this is partly due to our pair model and partly due to our reliance on traditional sequence alignment techniques. Edit-distance–based methods, on the other hand, are specialized for almost-perfect matches, which basically restricts their use on short and less erroneous reads. Recent advances in sequencing technologies suggest the contrary—while reads are getting longer, they still remain error prone.

## Supplementary Material

Supplementary Data
