# Supplementary material for: An approximate Bayesian approach for mapping paired-end DNA reads to a reference genome
Source: Bioinformatics. 2013 Feb 14;29(8):965–72. doi: 10.1093/bioinformatics/btt073 (PMC3624798; doi:10.1093/bioinformatics/btt073)
Supplement: Supplementary Data [file supp_29_8_965__index.html]

An approximate Bayesian approach for mapping paired-end DNA reads to a reference genome — An approximate Bayesian approach for mapping paired-end DNA reads to a reference genome — Supplementary Data 

# An approximate Bayesian approach for mapping paired-end DNA reads to a reference genome

## Supplementary Data

files

**Files in this Data Supplement:**

- Supplementary Data - pdf file
