# Supplementary material for: An approximate Bayesian approach for mapping paired-end DNA reads to a reference genome
Source: Bioinformatics. 2013 Feb 14;29(8):965–72. doi: 10.1093/bioinformatics/btt073 (PMC3624798; doi:10.1093/bioinformatics/btt073)
Supplement: Supplementary Data [file supp_btt073_supplementary-material.pdf]

An Approximate Bayesian Approach to Mapping Paired-End DNA Reads to a Reference Genome: Supplementary Material

Anish Man Singh Shrestha and Martin C. Frith

Computational Biology Research Center, AIST, Tokyo

1 Version information of aligners tested

Last	193
BWA	0.6.1
Bowtie	2.0.0-beta5
GSNAP	2012-06-20
Novoalign	V2.07.18
SOAP	2.21
Stampy	1.0.16

2 Simulation workflow

2.1 Simulating a genome

We used the `fasta-polymorph` program from the DNemulator package (www.cbrc.jp/dnemulator) to simulate a diploid human genome by incorporating polymorphisms from snp132Common.txt into the UCSC human genome hg19 as follows:

```
fasta-polymorph -a simulated_genomeVSreference.seg snp132Common.txt \
    hg19.fa hg19.fa > simulated_genome.fa
```

The `-a` switch in the command above records the alignments between the new simulated reference and hg19 in the file `simulated_genomeVSreference.seg`. This is required since snp132Common.txt also contains indel polymoprhisms. The "seg" format is used by Seg-suite (www.cbrc.jp/seg-suite), a package we used for evaluating mapping results. Using `hg19.fa` twice simulates a diploid genome.

2.2 Simulating a set of paired-end reads

Next from the simulated genome, we randomly sampled 1 million random fragments with size normally distributed with mean `FRAGMENT_SIZE_MEAN` and standard deviation `FRAGMENT_SIZE_SD`; and from each fragment, we obtained a pair of reads `READ_LENGTH` long:

```
fasta-paired-chunks -n 1000000 -l READ_LENGTH -f FRAGMENT_SIZE_MEAN -s FRAGMENT_SIZE_SD \
    simulated_genome.fa simulated_reads1.fa simulated_reads2.fa
```

Using the `fastq-sim` program in DNemulator, we simulated sequencer errors by using the per-base error probability values from fastq files of real datasets:

```
fastq-sim simulated_reads1.fa real_reads_1 > simulated_reads1.fastq
fastq-sim simulated_reads2.fa real_reads_2 > simulated_reads2.fastq
```

The following four datasets were constructed using this method, all of which are publicly available at `www.cbrc.jp/~anish/paired-end-benchmark`.

Simulated Dataset	READ_LENGTH	FRAGMENT_SIZE_MEAN	FRAGMENT_SIZE_SD	Error profile from:
1	100bp	288	27	ERR037752
2	100bp	288	27	SRR067577
3	76bp	200	40	ERR007826
4	35bp	160	30	ERR000408

2.3 Evaluating mapping results

In order to decide the correctness of mappings reported by each aligner, we used the Seg-suite package. First we computed the true alignment of each simulated read to the reference. For example, to compute this for the reads in `simulated_reads1.fa`, we executed the following:

```
awk '/>/ {print $3, $4, $5, $2, $6 == "+" ? 0 : -$3}' simulated_reads1.fa | \
  seg-sort > simulated_reads1VSsimulated_genome.seg

seg-swap simulated_genomeVSreference.seg | seg-sort > refgenomeVSsimulated_genome.seg

seg-join refgenomeVSsimulated_genome.seg simulated_reads1VSsimulated_genome.seg |cut -f1,4-7 | \
   seg-sort > true_alignments1.seg
```

The first command computes the alignments between the simulated reads and the simulated genome by using the read header information in the fasta file generated by `fasta-paired-chunks`. The second command prepares the alignments recorded in `simulated_genomeVSreference.seg` for the next command. The third command compares the alignments in the two seg files to obtain the true alignments between the reads and the reference.

Next the alignments predicted by the aligners were converted to seg format. The following example is for the sam format, which is the format used by most of the aligners

```
seg-import sam predicted_alignments.sam | seg-sort > predicted_alignments.seg
```

Finally, we counted the reads that were mapped correctly. A mapping was considered correct if the predicted alignment and the true alignment matched at, at least one position. To compute the number of reads in `simulated_reads1.fastq` that were correctly aligned, we executed the following:

```
seg-join -w true_alignments1.seg predicted_alignments.seg |cut -f4 |sort -u| wc -l
```

3 Aligner settings corresponding to the figures in the main article

With Last, we used the following commands for all of the tests. These are the recommended parameters in the Last documentation.

```
lastdb -m1111110 index reference_genome
lastal -Q1 -d108 -e120 -i1 index reads1 > out1.maf
lastal -Q1 -d108 -e120 -i1 index reads2 > out2.maf
last-pair-probs.py -m 0.1 out1.maf out2.maf > result.maf
```

Execution commands corresponding to Figures 1, 3(a), 4, and 5 of the main article are shown below. For aligners that require the user to input fragment length information, we used 288 $\pm$ 135, which is equivalent

to 5 standard deviations about the mean fragment length used in the generation of our simulated 100bp dataset.

Novoalign	`novoindex index reference_genome`<br>`novoalign -Q 6 --Q2off -i PE 288,27 -d index -f reads1 reads2 > result`
Stampy	`stampy.py -G index reference_genome`<br>`stampy.py -g index -H index`<br>`stampy.py --minposterior=6 -g index -h index -M reads1 reads2 > result`
BWA	`bwa index -a bwtsw -p index reference_genome`<br>`bwa aln index reads1  > out1.sai`<br>`bwa aln index reads2  > out2.sai`<br>`bwa sampe index out1.sai out2.sai reads1 reads2 > result`
Bowtie	`bowtie2-build reference_genome index`<br>`bowtie2 -x index -1 reads1 -2 reads2 -I 153 -X 423 --local --very-sensitive-local -S result`
GSNAP	`gmap_build -D index reference_genome`<br>`gsnap -D index_dir -d index reads1 reads2 --pairexpect=288 --pairdev=135 -A sam > result`
SOAP	`2bwt-builder reference_genome`<br>`soap -a reads1 -b reads2 -D index -o result-paired -2 result-unpaired -m 153 -x 423 -r 0`

Execution commands corresponding to Figure 3(b) of main article are shown below. For aligners that require the user to input fragment length information, we used 200 ± 160, which is equivalent to 4 standard deviations about the mean fragment length used in the generation of our simulated 76bp dataset.

Novoalign	`novoalign -Q 6 --Q2off -i PE 200, 40 -d index -f reads1 reads2 > result`
Stampy	`stampy.py --minposterior=6 -g index -h index -M reads1 reads2 > result`
BWA	`bwa aln index reads1  > out1.sai`<br>`bwa aln index reads2  > out2.saiq`<br>`bwa sampe index out1.sai out2.sai reads1 reads2 > result`

Bowtie	`bowtie2 -x index -1 reads1 -2 reads2 -I 40 -X 360 --very-sensitive -S result`
GSNAP	`gsnap -D index_dir -d index reads1 reads2 --pairexpect=200 --pairdev=160 -A sam > result`
SOAP	`soap -a reads1 -b reads2 -D index -o result-paired -2 result-unpaired -m 40 -x 360 -r 0`

Execution commands corresponding to Figure 3(c) are shown below. For aligners that require the user to input fragment length information, we used 160 ± 150 which is equivalent to 5 standard deviations about the mean fragment length used in the generation of our 35bp dataset. The same settings were also used in the experiment in Section 3.6/ Figure 6 of the main article.

Novoalign	`novoalign -Q 6 --Q2off -i PE 160,30 -d index -f reads1 reads2 > result`
Stampy	`stampy.py --minposterior=6 -g index -h index -M reads1 reads2 > result`
BWA	`bwa aln index reads1  > out1.sai`<br>`bwa aln index reads2  > out2.saiq`<br>`bwa sampe index out1.sai out2.sai reads1 reads2 > result`
Bowtie	`bowtie2 -x index -1 reads1 -2 reads2 -I 10 -X 310 --very-sensitive -S result`
GSNAP	`gsnap -D index_dir -d index reads1 reads2 --pairexpect=160 --pairdev=150 -A sam > result`
SOAP	`soap -a reads1 -b reads2 -D index -o result-paired -2 result-unpaired -m 10 -x 310 -r 0`

4 Parameter testing for select aligners

For some of the aligners, we arrived at the above settings by trying out several combinations of parameters and choosing the ones that provided a good combination of speed and accuracy. We describe below our trials for Bowtie, BWA, and SOAP.

4.1 Bowtie

Bowtie can do an end-to-end (align the whole of the read to a region of the reference) or a local alignment. For both modes, Bowtie2 provides a very handy set of parameter presets to control sensitivity/speed. For

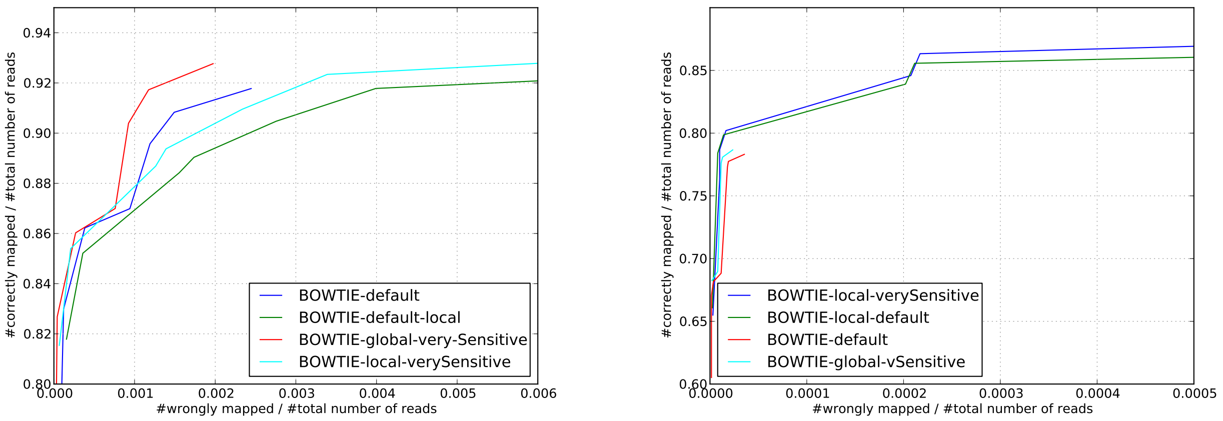

(a)35bp (b) 100bp

Figure 1: Testing Bowtie parameters

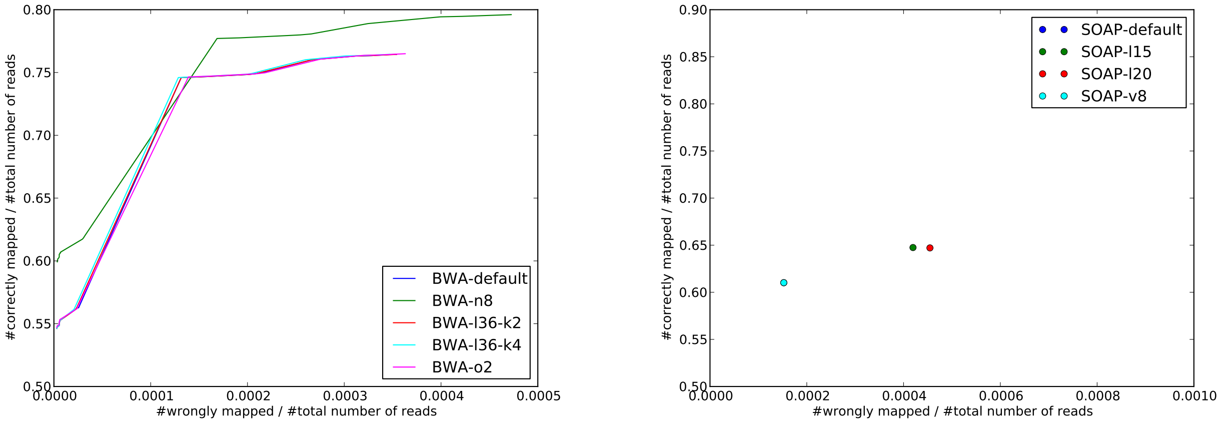

(a) (b)

Figure 2: Testing (a) BWA and (b) SOAP parameters. In (b), the points for SOAP-default and SOAP-v8 coincide.

our datasets, we tried both modes and various presets. We show results for some settings in Figure 1. We chose `--local --verySensitive-local` for 100bp reads and `--verySensitive` in the end-to-end mode for 76bp and 35bp reads.

4.2 BWA

With BWA, we tried varying the following parameters: (1) `n` which controls the maximum edit distance (2) `l` which controls the length of initial portion of the read which to be taken as seed (3) `k` which controls the maximum differences allowed in the seed, and (4) `o` which controls the maximum number of gap openings allowed. We show some of the results in Figure 2(a). We observed that increasing `n` improves the sensitivity, but this comes at an enormous speed cost. Changing the other parameters did not bring about any significant performance boost, therefore we settled for default settings throughout all our tests.

4.3 SOAP

With SOAP, we tried varying the following parameters: (1) `v` that controls the maximum edit distance (2) `l` which controls the length of the initial portion of the read which is to be used as seed. Suprisingly, varying

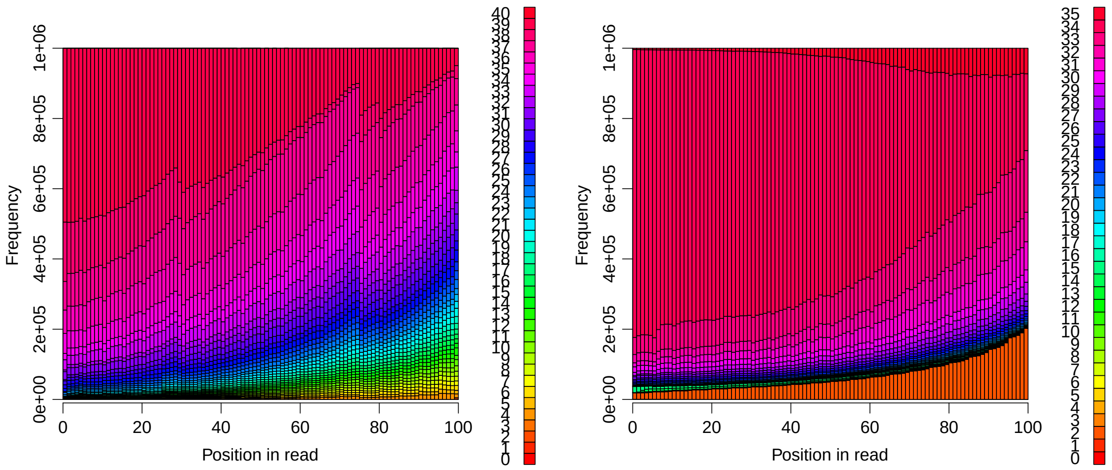

Figure 3: The error profiles of our two datasets of 100bp-long reads: (a) the dataset generated from ERR037752 used in the main article and (b) the second dataset generated from SRR067577.

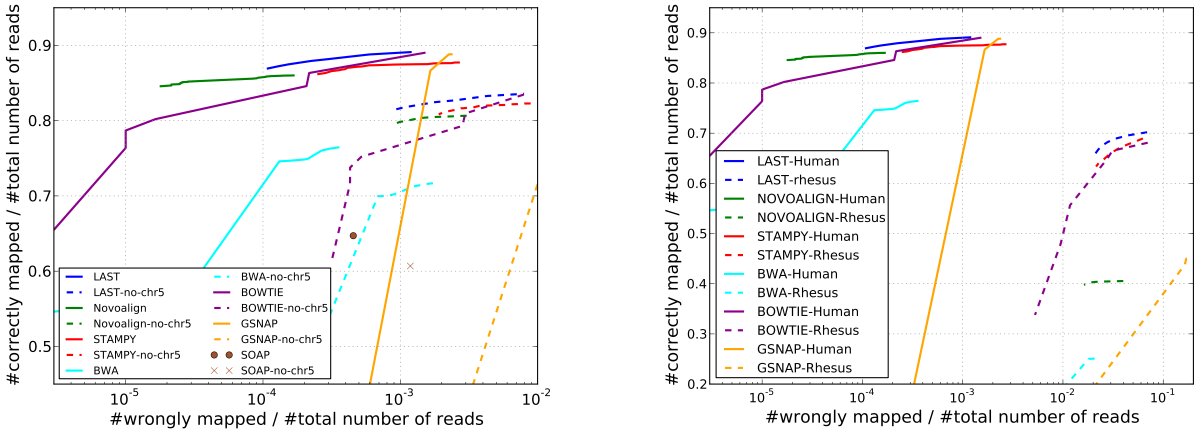

(a) (b)

Figure 4: (a) Mapping to the hg19 (solid) and hg19 with missing chr5 (dashed). (b) Mapping to the rhesus genome.

v had no effect. Varying l slightly improves sensitivity, but drastically reduces speed. Therefore, we settled with default values in all our tests.

5 Effect of error profile: a different dataset

To understand the effect of error profile on aligner performance and to make our results more robust, we repeated the experiments in Section 3.2 through Section 3.5 on our second 100bp-read dataset, which was generated using the same random fragments but simulating the sequencing error based on error probabilities from SRR067577. We can see from Figure 3 that the new dataset has significantly more low-quality reads. The results of mapping this new set of reads to the human reference hg19 (with and without Chromosome 5) and to the rhesus genome rheMac2 are shown in Figure 4. The result of mapping the reads after shuffling the same 40% of the read pairs as in the chromosomal translocations experiment in the main article is shown in Figure 5. Results similar to the ones in the main article were observed.

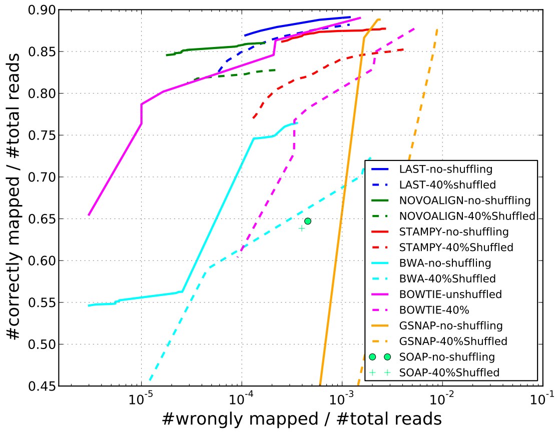

Figure 5: Mapping shuffled read pairs.

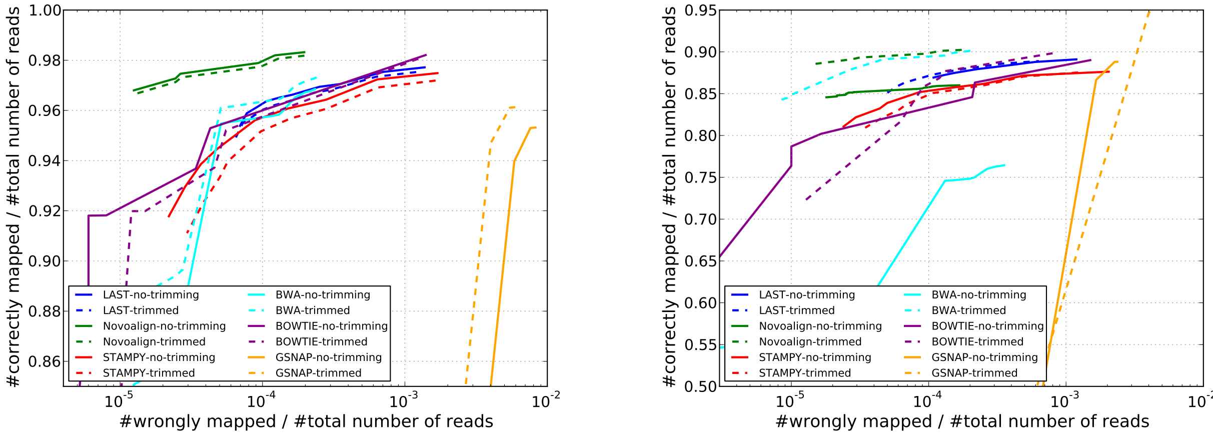

(a) (b)

Figure 6: Comparing the performance with and without read trimming (dashed and solid lines, respectively).

6 Effect of trimming

To deal with low quality positions towards the 3' end of a read, which is typical of some of the widely-used sequencing platforms, some aligners provide an option to trim the reads. None of them use trimmming in their default settings. Last uses sequence quality information during alignment and has no mismatch limit, and therefore it does not require explicit trimming of reads. To understand the effect of trimming, we applied BWA-style trimming (using DynamicTrim of the SolexaQA package with a threshold of $p = 0.05$) to our two 100bp datasets – the error profiles of which can be seen in Figure 3 – and compared the results of mapping trimmed and untrimmed reads (Figure 6). As can be seen from Figure 3, the dataset generated using quality values from SRR067577 has a higher number of reads with low quality scores, especially towards the 3' position; and it is not surprising that trimming this dataset vastly improves the accuracy of aligners like BWA. Last and Stampy are not affected much by trimming. On the contrary, their performance drops slightly with trimming, likely due to loss of information that comes from trimming.

These datasets are especially conducive to trimming because they have few medium-quality bases. The edit-distance-centric aligners do well when there are very few errors, which includes the case where a read ends with uninformative very-low quality bases that are removed by trimming. Last would presumably be

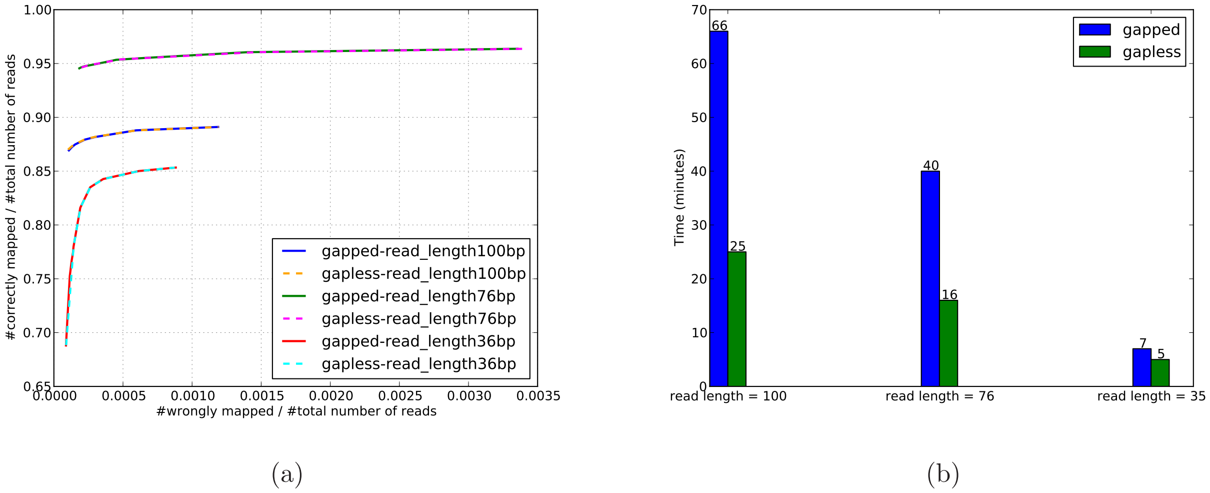

Figure 7: (a) No loss in accuracy was observed for gapless alignments of 3 sets of 1 million pairs of human reads with varying lengths to the human reference. (b) Time taken for execution on a single core of a machine with Xeon X5680(3.3GHz/6core) processor and 60GB RAM (please note that this machine is different from the one used to measure the running times shown in Fig. 3(d) of the main article).

favored when there are many medium-quality bases, which boost the error rate, but convey information that would be lost by trimming. It should also be pointed out that there exist datasets in which low quality bases are not confined to the ends. Trimming is not a solution for such cases, but for Last they pose no particular problems.

7 Last speed-up

7.1 Gapless alignment

Last performs gapless alignments significantly faster than gapped alignments. Mapping our 3 datasets of varying read lengths to the human reference in both gapped and gapless mode, we observed that gapless alignment results in no loss in sensitivity while the running time, not including the pairing phase, drops by more than 50% for longer reads (Figure 7).

7.2 Using a smaller file format

Since our paired-end module is written in Python, file I/O can be time consuming. By changing the ouptut format of the alignment phase to a leaner file format, we can reduce the running time of the pairing phase by almost 30 % (see Figure 8).

8 Memory usage

Memory usage is dominated by the index of the reference genome which is loaded into memory. With easy availability of ever-increasing memory, none of the aligners pose any serious problems.

Aligner	Index type	Size
Last	suffix array + auxiliary table	15.0GB
Novoalign	hash table	7.7GB

Stampy	hash table	2.8 GB
BWA	BWT	5.0GB
Bowtie	BWT	3.8GB
GMAP	hash table	5.0GB
SOAP	BWT	5.9 GB

9 Effect of prior probability

Last allows the user to set the value of the prior probability that a read pair is disjoint. It is possible to estimate this probability from the set of reads that map uniquely (However, we have not implemented this feature yet). Depending on the input, estimating this value rather than using the default of 0.01 can improve mapping accuracy. To test this, we reran Last on our shuffled dataset (used in the experiment simulating chromosomal translocations), with the disjoint probability set to exactly the ratio of shuffled reads (0.4). The results are shown in Figure 9. A similar trial with Stampy, in which we changed a similar setting provided by Stampy proved ineffective.

10 The "mapping percentage" metric

Many papers use the percentage of reads mapped to evaluate and compare the performance of mapping tools. While it can be a helpful metric, we do not explicitly compare this figure for various reasons. First, if necessary this information can be easily worked out from the curves in Figure 3 of the main text. Secondly, even for a single run of an aligner, the percentage of reads mapped varies depending on the what mapping quality threshold is set. For example, the table below shows for a run of Last the percentage of reads mapped for various mismap probability values. As we relax the threshold stringency, we get greater mapping percentage because we are allowing more lower-quality mappings. For this reason, comparison based on mapped percentage can be misleading. Finally, it is a trivial task to build an aligner that always maps a 100 percent of the reads. Therefore it should be the correctness of the mappings we should be concerned about.

mismap probability less than	percentage of reads mapped
0.1	97.85
0.2	97.90
0.3	97.95
0.4	97.99
0.5	98.05
0.6	98.94
0.7	99.23
0.8	99.38
0.9	99.56

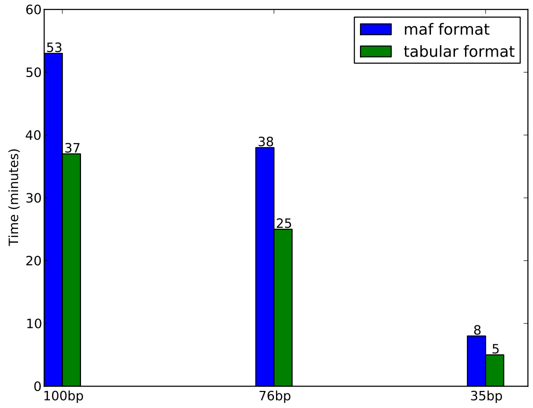

Figure 8: Comparing running times of pairing phase when using the maf and tabular format, on a single core of a machine with Xeon X5680(3.3GHz/6core) processor and 60GB RAM (please note that this machine is different from the one used to measure the running times shown in Fig. 3(d) of the main article).

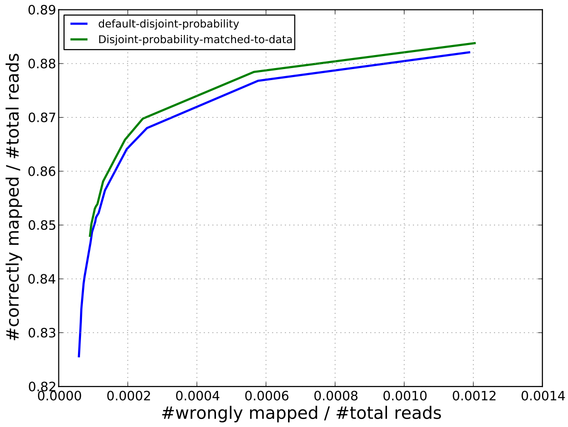

Figure 9: Mapping accuracy improves by changing the prior disjoint probability.
